# Supplementary material for: A Simplified Perchloric Acid Workflow With Neutralization (PCA N) for Democratizing Deep Plasma Proteomics at Population Scale
Source: Mol Cell Proteomics. 2025 Sep 19;24(11):101071. doi: 10.1016/j.mcpro.2025.101071 (PMC12597270; doi:10.1016/j.mcpro.2025.101071)
Supplement: Supplemental Data [file mmc1.pdf]

A simplified perchloric acid workflow with neutralization (PCA-N) for democratizing deep plasma proteomics at population scale

Vincent Albrecht†, Johannes B. Müller-Reif†, Vincenth Brennsteiner, Matthias Mann*

† These authors contributed equally

* To whom correspondence should be addressed mmann@biochem.mpg.de

Fig. S1. PCA-N workflow enables highly parallelized sample preparation.

Fig. S2. Mass spectrometry method optimization for deep plasma proteome coverage.

Fig. S3. Long term repeatability assessment of PCA-N and NEAT workflows according to the CLSI C64 guideline.

Fig. S4. Technical validation of the PCA-N workflow in an extreme-scale plasma proteomics study.

Supplemental Data 1. Protein quantification for the NEAT workflow.

Supplemental Data 2. Protein quantification for the PCA-N workflow.

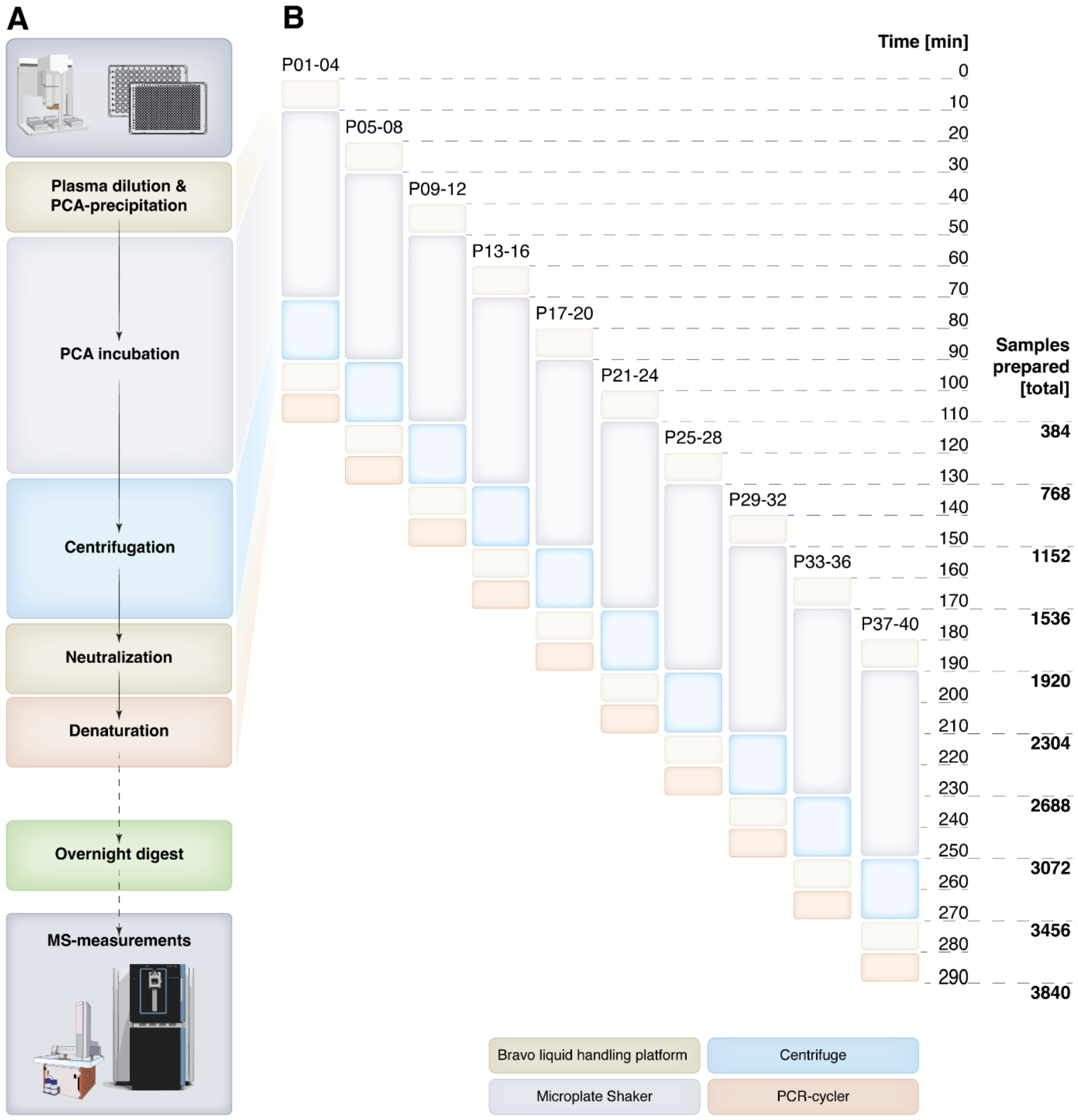

FIG. S1. **PCA-N workflow enables highly parallelized sample preparation. A.** Schematic representation of the PCA-N workflow with main processing steps shown in sequential order from plasma sample to mass spectrometry analysis. **B.** Timeline illustrating the parallelized sample preparation strategy. The workflow leverages staggered use of liquid handling systems, centrifuges and PCR-cycler to efficiently process multiple plates simultaneously. This example demonstrates the processing of 3840 plasma samples (40 96-well plates in batches of 4 plates) in under 300 min, with cumulative sample throughput indicated on the right (384, 768, 1152, etc.).

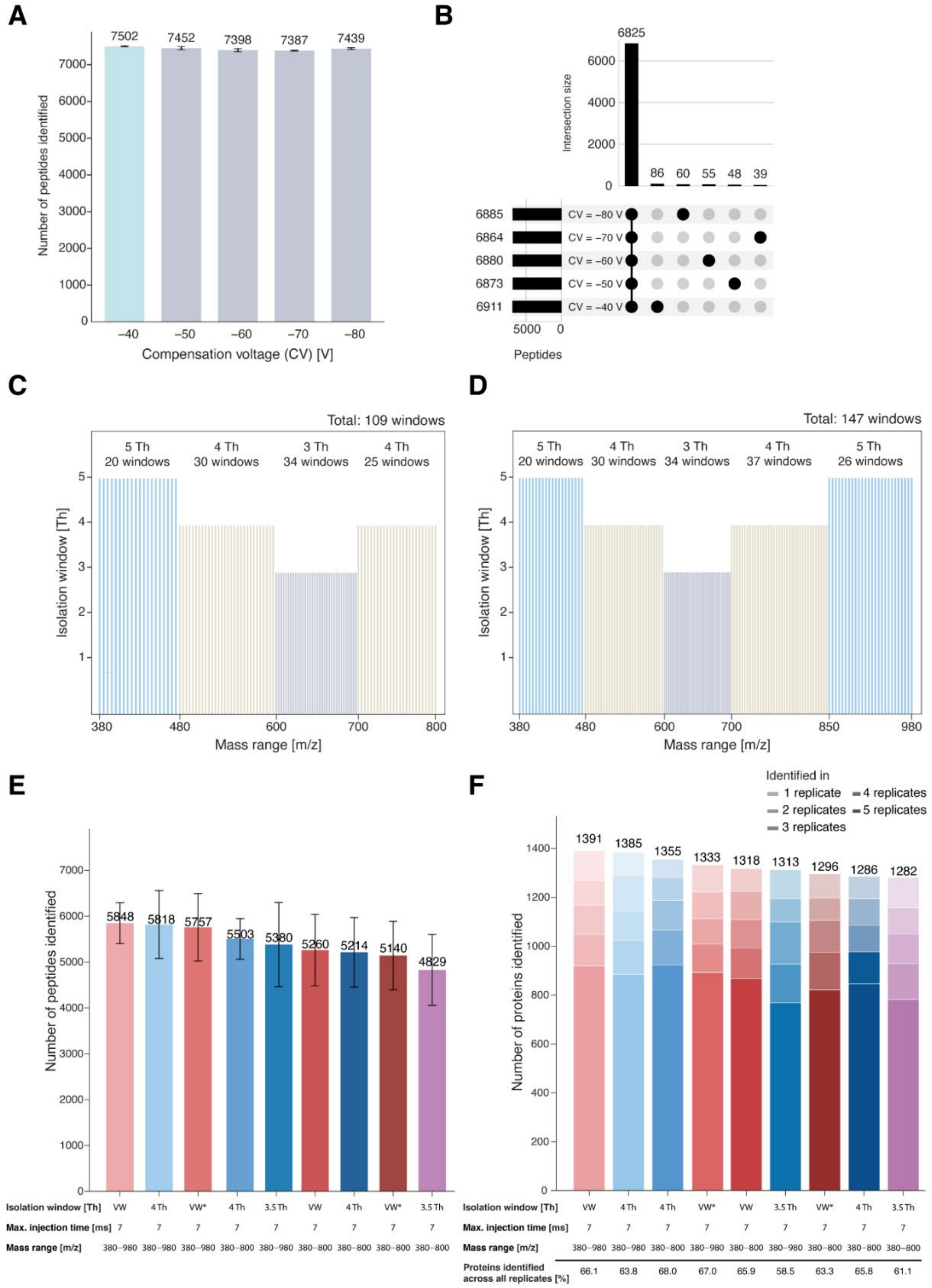

FIG. S2. **Mass spectrometry method optimization for deep plasma proteome coverage. A.** Number of peptide identifications using different FAIMS compensation voltages (CV) ranging from -40 to -80 V. **B.** UpSet plot showing unique peptides per CV and the intersection of peptides shared across multiple CV settings. Other overlaps are not shown. **C.** Variable isolation window method with 5, 4, 3 and 4 Th windows spanning mass range 380-800 m/z (total 109 windows). **D.** Extended mass range variable isolation window method with 5, 4, 3, 4 and 5 Th windows spanning mass range 380-980 m/z (total 147 windows). **E.** Number of peptides identified using different MS methods comparing standard equally spaced windows and variable width windows (VW) (VW* = using optimal window placement). **F.** Number of proteins identified across 1 - 5 analytical replicates using different MS methods, with color intensity indicating the number of replicates in which proteins were detected. Numbers above bars indicate total proteins identified. Percentage value show identification rates across all replicates.

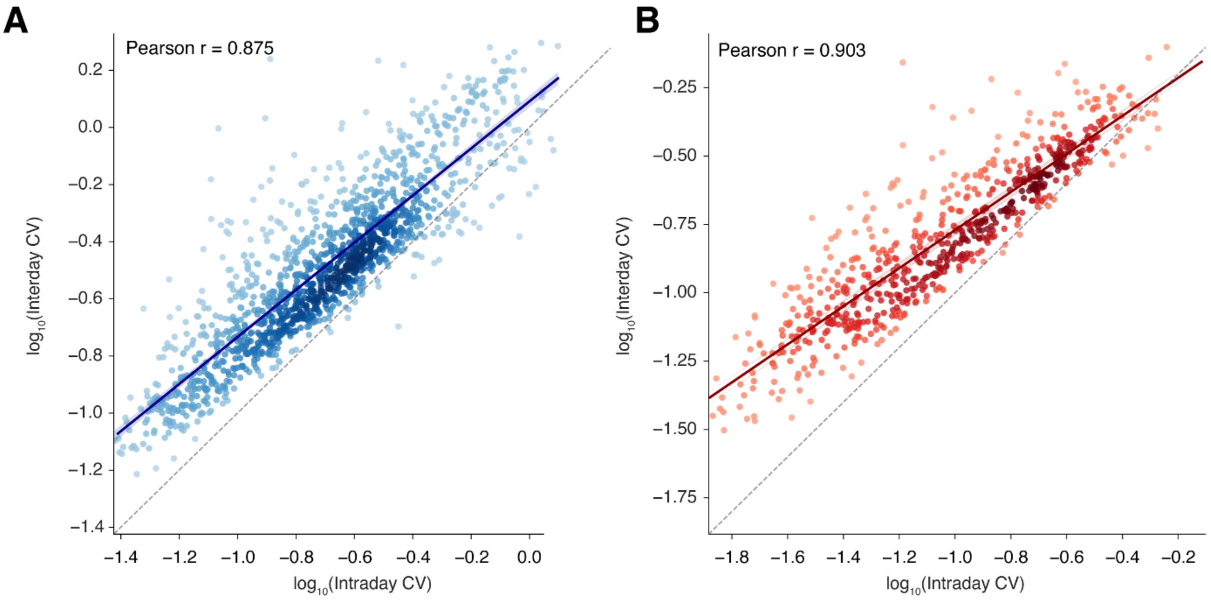

Fig. S3. **Long term repeatability assessment of PCA-N and NEAT workflows according to the CLSI C64 guideline.** Pearson correlation of $\log_{10}$-transformed intraplate and interplate CVs for PCA-N (**A**) and NEAT (**B**).

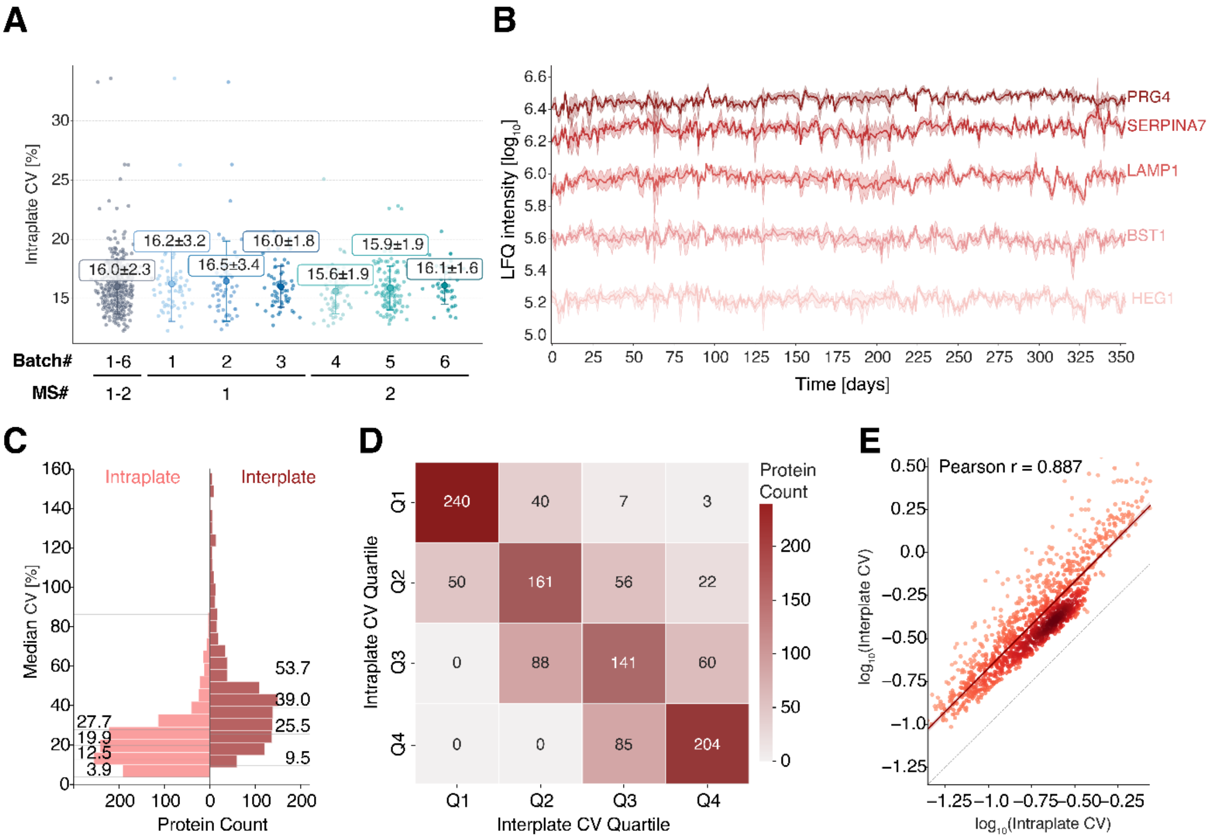

FIG. S4. **Technical validation of the PCA-N workflow in an extreme-scale plasma proteomics study.** Non-batch-corrected data is used for the following displays. **A.** Intraplate median coefficients of variation (CVs) for the combined dataset with 1,705 quality control plasma samples and split by the individual sample batches/cohorts. **B.** Reproducibility of the label-free quantification (LFQ) intensities of the top five proteins with the lowest CVs throughout the 353-day measurement period. The line represents the mean values and the shading the standard deviation for PRG4 (Proteoglycan 4) (CV_{PRG4} = 6.9%), SERPINA7 (Thyroxine-binding globulin) ($CV_{SERPINA7}$ = 8.8%), LAMP1 (Lysosome-associated membrane glycoprotein 1) (CV_{LAMP1} = 8.8%), BST1 (ADP-ribosyl cyclase/cyclic ADP-ribose hydrolase 2) (CV_{BST1} = 8.7%) and HEG1 (Protein HEG homolog 1) (CV_{HEG1} = 8.7%). **C.** Distribution of CV values for proteins ($N_{Proteins}$=1,157) in both intraplate (light red) and interplate (dark red) measurements. Numbers indicate median CVs [%] for each distribution quartile. **D.** Confusion matrices showing the stability of protein CV rankings between intraplate and interplate measurements. Numbers in each cell represent protein counts, with 64.5% of proteins maintaining the same quartile rankling (diagonal values). **E.** Pearson correlation of log_{10}-transformed intraplate and interplate CVs.

SUPPLEMENTAL DATA 1. **Protein quantification for the NEAT workflow.** This table contains median protein intensities for the NEAT workflow based on five technical replicates. Protein.Group: UniProt protein group identifier(s) derived from DIA-NN gene-level inference. Genes: Gene symbol(s) corresponding to each protein group. LFQ_Median: Median label-free quantification (LFQ) intensity for each protein across replicates.

SUPPLEMENTAL DATA 2. **Protein quantification for the PCA-N workflow.** This table contains median protein intensities for the PCA-N workflow based on five technical replicates. Protein.Group: UniProt protein group identifier(s) derived from DIA-NN gene-level inference. Genes: Gene symbol(s) corresponding to each protein group. LFQ_Median: Median label-free quantification (LFQ) intensity for each protein across replicates.
